# Low-energy total diet replacement intervention in patients with type 2 diabetes mellitus and obesity treated with insulin: a randomized trial

**DOI:** 10.1136/bmjdrc-2019-001012

**Published:** 2020-01-28

**Authors:** Adrian Brown, Anne Dornhorst, Barbara McGowan, Omar Omar, Anthony R Leeds, Shahrad Taheri, Gary S Frost

**Affiliations:** 1 Nutrition and Dietetic Research Group, Imperial College London, London, UK; 2 Centre for Obesity Research, Department of Medicine, University College London, London, UK; 3 Division of Diabetes, Endocrinology and Metabolism, Imperial College London, London, UK; 4 Institute of Diabetes, Endocrinology and Obesity, Guy’s and St Thomas’ Hospital, London, UK; 5 Department of Medicine and Clinical Research Core, Weill Cornell Medicine–Qatar, Qatar–Foundation Education City, Doha, Qatar; 6 Birmingham Clinical Trials Unit, University of Birmingham, Birmingham, UK; 7 Nutrition, Exercise and Sports, Faculty of Science, University of Copenhagen, Copenhagen, Denmark; 8 Clinical Research Unit, Parker Institute, Frederiksberg Hospital, Copenhagen, Denmark; 9 Department of Medicine, Weill Cornell Medical College, New York, New York, USA

**Keywords:** insulin, low calorie diet, obesity, type 2 diabetes

## Abstract

**Objectives:**

The management of patients with long-standing type 2 diabetes and obesity receiving insulin therapy (IT) is a substantial clinical challenge. Our objective was to examine the effect of a low-energy total diet replacement (TDR) intervention versus standardized dietetic care in patients with long-standing type 2 diabetes and obesity receiving IT.

**Research design and methods:**

In a prospective randomized controlled trial, 90 participants with type 2 diabetes and obesity receiving IT were assigned to either a low-energy TDR (intervention) or standardized dietetic care (control) in an outpatient setting. The primary outcome was weight loss at 12 months with secondary outcomes including glycemic control, insulin burden and quality of life (QoL).

**Results:**

Mean weight loss at 12 months was 9.8 kg (SD 4.9) in the intervention and 5.6 kg (SD 6.1) in the control group (adjusted mean difference −4.3 kg, 95% CI −6.3 to 2.3, p<0.001). IT was discontinued in 39.4% of the intervention group compared with 5.6% of the control group among completers. Insulin requirements fell by 47.3 units (SD 36.4) in the intervention compared with 33.3 units (SD 52.9) in the control (−18.6 units, 95% CI −29.2 to –7.9, p=0.001). Glycated Hemoglobin (HbA1c) fell significantly in the intervention group (4.7 mmol/mol; p=0.02). QoL improved in the intervention group of 11.1 points (SD 21.8) compared with 0.71 points (SD 19.4) in the control (8.6 points, 95% CI 2.0 to 15.2, p=0.01).

**Conclusions:**

Patients with advanced type 2 diabetes and obesity receiving IT achieved greater weight loss using a TDR intervention while also reducing or stopping IT and improving glycemic control and QoL. The TDR approach is a safe treatment option in this challenging patient group but requires maintenance support for long-term success.

**Trial registration number:**

ISRCTN21335883.

**Significance of this study**

**What is already known about this subject?**

- The management of patients with obesity and type 2 diabetes treated with insulin is clinically challenging because while insulin treatment improves glycemic control, it exacerbates excess body weight and is associated with reduced quality of life.
- The Diabetes in Remission Clinical Trial showed that a total diet replacement program using a formula low-energy diet results in diabetes remission in about half of those recently diagnosed with type 2 diabetes. The Doctor Referral of Overweight People to Low Energy Total Diet Replacement Treatment trial further demonstrated the successful implementation of total diet replacement program in primary care. No randomized controlled trial, however, has examined the use of a formula low-energy diet total diet replacement program in those with low-energy diet concurrently treated with insulin.

**What are the new findings?**

- Our study demonstrates the effectiveness and safety of a low-energy diet total diet replacement program intervention in those with long-standing type 2 diabetes and obesity treated with insulin.
- The total diet replacement program intervention resulted in a significant reduction in body weight compared with standardized dietetic care (9.8 kg vs 5.6 kg, respectively) and insulin burden (39.4% vs 5.6% stopping insulin therapy) and improved quality of life at 12 months.

**How might these results change the focus of research or clinical practice?**

- These results change the focus of clinical practice by demonstrating that low-energy formula diets are an effective clinical option in the management of patients with long-standing type 2 diabetes treated with insulin.

## Introduction

Type 2 diabetes mellitus is perceived as a progressive disease with continuous pancreatic beta-cell dysfunction necessitating insulin therapy (IT) within 10 to 12 years of diagnosis.^1 2^ IT improves glycemic control and reduces microvascular complications, but does not significantly benefit cardiovascular disease (CVD) morbidity and mortality.^1 3 4^ IT is associated with adverse outcomes including weight gain, hypoglycemia, and reduced quality of life (QoL).^5^

Bariatric surgery results in significant IT reduction in patients with type 2 diabetes and obesity with approximately 90% discontinuing IT at 5 years.^6^ Energy restriction is a key mechanism for the beneficial metabolic effects of bariatric surgery.^7^ Bariatric surgery, however, is not appropriate or acceptable for all patients, has a number of complications, and economic constraints limit its availability. Unlike bariatric surgery, traditional dietary interventions do not achieve long-term reduction in IT therapy burden.^8 9^

Total diet replacement (TDR) using a formula low-energy diet (LED) program, to replicate the energy deficit and weight loss through bariatric surgery, promotes type 2 diabetes remission in those recently diagnosed with type 2 diabetes.^10^ Few studies, however, have rigorously examined the use of a formula LED TDR in those with long-standing type 2 diabetes receiving IT, limiting their value to inform clinical guidelines and practice.^11–14^ Therefore, we undertook a randomized clinical trial comparing the impact of a TDR intervention including a formula LED, behavior modification and physical activity, with standardized dietetic care on weight loss, insulin burden and glycemic control in patients with type 2 diabetes and obesity receiving IT.

## Methods

### Study design and participants

We conducted a prospective, parallel-group, non-blinded randomized clinical trial in two hospitals in London, UK (Imperial College Healthcare National Health Service (NHS) Trust and Guy’s and St Thomas’ NHS Foundation Trust). Participants with type 2 diabetes and obesity treated with IT were identified and recruited from UK primary and secondary care. Participants had type 2 diabetes, were treated with insulin, had a body mass index (BMI) of ≥30 kg/m^2^, were aged 18–70 years and provided written informed consent. Key exclusion criteria included being on IT for >10 years with a fasting circulating C-peptide of less than 600 pmol/L, type 1 diabetes, significant diabetes microvascular complications, estimated glomerular filtration rate of less than 30 mL/min/1.73 m^2^ and clinically diagnosed with binge eating disorder. The supplementary appendix has details on full eligibility and withdrawal criteria and recruitment methods.

### Randomization and masking

Randomization was performed using an online software tool (Sealed Envelope) to either the intervention or control group in a 1:1 ratio using computer-generated random numbers. To ensure group balance (age, gender, ethnicity, diabetes duration) minimization was used.^15^ A 30% chance of simple random allocation was included.^16^ Due to the nature of the interventions participants could not be blinded so were aware of the group allocation; however, the study statistician (OO) was blinded to allocation for analysis.

### Dietary interventions

At randomization, participants commenced a 12-week TDR formula LED (Cambridge Weight Plan, Northants, UK) followed by 12 weeks of structured food reintroduction and then ongoing follow-up in combination with an energy deficit diet at 3-month intervals until 12 months. For the first 12 weeks, all meals were replaced with four formula LED products per day (800–820 kcal/day, 57% carbohydrate, 14% fat, 26% protein and 3% fiber) in addition to at least 2.25 liters of energy-free beverages. A fiber supplement was recommended, if required, to avoid constipation, a common side effect of using a TDR.

### Standardized dietetic care

Participants followed a standardized weight management program using a 600 kcal deficit diet for 12 months, aiming for weight loss of 0.5–1.0 kg/week, based on current national guidelines.^17^ This was based on total energy expenditure estimated from their basal metabolic rate using the Mifflin St-Jeor equation^18^ and physical activity levels (online supplementary figure S1).

10.1136/bmjdrc-2019-001012.supp1 Supplementary data

### Subject counseling support

Both groups were seen by the same specialist dietician after 1 week and then monthly for the first 6 months (eight face-to-face sessions of 30–60 min), in addition to seven telephone consultations of 15–20 min in between. The maintenance phase matched standard type 2 diabetes healthcare provision with two face-to-face sessions from 6 to 12 months. Participants received behavioral support to aid lifestyle adherence and maintenance^19 20^ and were encouraged to undertake moderate exercise, as per guidelines, of at least 30 minutes, 5 days per week including both aerobic and resistance exercise.^17^ QoL was measured using EuroQol-5 Dimension. Participants in both groups received concomitant standard diabetes care based on UK national guidelines.^21^

### Insulin titration

Insulin was titrated by algorithm to ensure glycemic control and safety. At randomization, insulin dose was reduced by 50% in the intervention group and 30% in the control group. Further adjustments were made according to glycemic control, intervention allocation and non-insulin medications. Participants were advised to perform home capillary blood glucose monitoring (4–6 times/day) to inform insulin titration. If hypo/hyperglycemia occurred, insulin was adjusted to maintain glycemic targets (online supplementary figure S2). At the start of both the intervention and control, glucagon-like peptide-1 (GLP-1) receptor agonists and sodium-glucose cotransporter-2 (SGLT-2) inhibitors were stopped as these had the potential to impact on body weight, which was the primary outcome.

### Procedures

Data were collected at baseline, 3, 6 and 12 months. Anthropometric measurements were made with participants barefoot wearing light clothing. Weight and body composition (using bioelectrical impedance) were measured to the nearest 0.1 kg following a 12-hour overnight fast (MC-780MAP, Tanita UK, Middlesex, UK). Height was measured to the nearest 0.1 cm in the morning of screening, using a stadiometer (Invicta Plastics, Leicester, UK). Hip and waist circumference was measured to the nearest 0.1 cm using a fiberglass tape.

Mixed meal tolerance tests (MMTT) were undertaken at baseline, 3 and 12 months. Following an overnight fast, a cannula was inserted into the subject’s forearm vein and fasted samples taken. A standardized liquid supplement was given (Ensure Plus, Abbott, UK; 330 calories: 54% carbohydrate, 17% protein, and 29% fat) and timed blood samples collected (15, 30, 45, 60, 90, 120, 150, 180, 210 min after test meal). Appetite and hunger were assessed at each time point using a 100 mm visual analog scale, but are not reported.^22^ Fasting and postprandial plasma glucose, insulin and total GLP-1 were measured (see online supplementary appendix).

### Study outcomes

The primary outcome was weight loss at 12 months. Prespecified secondary outcomes included insulin usage, HbA1c, fasting plasma glucose, fasting plasma C-peptide, hormonal responses during the MMTT, serum lipids, blood pressure, body composition, and QoL. Post hoc outcomes examined differences between participants in the intervention group who discontinued IT and those who did not. Adverse events (AE) were monitored during the initial TDR and food reintroduction phase.

### Statistical analysis

The primary outcome was body weight loss at 12 months. Primary analysis used repeated measures analysis of covariance using a mixed model taking account of the within-subject variability, using weight measurements at all postrandomization time points and adjusting for baseline weight, randomization factors, HbA1c and number of medications. The adjusted mean group differences for baseline and each time point with 95% CIs were calculated. Both the crude unadjusted and adjusted estimates are presented, but the primary inference was based on the adjusted analysis. Area under the curve (AUC) was calculated using the trapezoidal rule. Secondary outcomes were analyzed using similar methods. Difference in hypoglycemia frequency between groups used mixed-effects Poisson regression with incidence rate ratio presented. All analyses were according to intention-to-treat principle. The impact of non-response and missing data at 12 months’ follow-up was examined in sensitivity analyses (online supplementary table S1). Details of dealing with missing data are found in the online supplementary appendix. Demographic factors and clinical characteristics were summarized with counts (percentages) for categorical variables, mean (SD) for normally distributed continuous variables, or median (IQR or entire range) for other continuous variables.

The sample size calculation indicated that 37 participants per group would provide 80% power to detect a 10 kg weight loss difference between groups (SD 15 kg) at a 5% significance level.^23^ Published mean attrition while following a very-low-energy diet (VLED) and LED is 0%–52%.^24^ Accounting for an approximate 20% dropout, a total of 90 participants (45 per arm) were recruited.

The analysis plan did not correct for multiple comparisons for tests of secondary outcomes. Results are reported as point estimates and 95% CIs. The CI widths were not adjusted for multiple comparisons, so intervals cannot infer definitive treatment effects. Statistical analyses were performed using Stata SE V.15.0 (StataCorp, College Station, TX). Statistical significance was defined as a two-sided p value <0.05.

## Results

Between 4 November 2014 and 19 June 2017, ninety participants were randomly assigned to the treatment groups (intervention, n=45; control, n=45; online supplementary figure S3). Follow-up ended on 29 May 2018. At baseline, participants in the study groups were matched for demographic, anthropometric and clinical characteristics (table 1). Overall, 21 (23.3%) participants were lost to follow-up or had withdrawn at 12 months, with 12/45 (26.7%) participants in the intervention group and 9/45 (20%) participants in the control group with no obvious differences in withdrawal reason between groups (online supplementary figure S3).

**Table 1:** Baseline characteristics of the patient population

Characteristics	Intervention (n=45)	Control (n=45)
Age at randomization, median (IQR)	58.5 (50.1–64.2)	56.1 (51.0–64.5)
Sex, n (%)		
Male	20 (44.4)	19 (42.2)
Female	25 (55.6)	26 (57.8)
Ethnicity, n (%)		
Caucasian	26 (57.8)	27 (60.0)
Mixed	1 (2.2)	1 (2.2)
Asian	4 (8.9)	7 (15.6)
Black	14 (1.1)	10 (22.2)
Weight (kg), mean (SD)	104.0 (20.2)	103.1 (18.9)
Body mass index (kg/m²), mean (SD)	36.6 (5.1)	36.8 (5.3)
Waist circumference (cm), mean (SD)	120.3 (12.7)	121.5 (12.4)
Hip circumference (cm), mean (SD)	120.7 (12.1)	122.0 (13.2)
Waist-to-hip ratio, mean (SD)	1.00 (0.06)	1.00 (0.06)
Body fat (%), mean (SD)	40.4 (8.0)	40.4 (7.4)
HbA1c (%), mean (SD)	8.7 (1.7)	9.3 (1.7)
Duration of diabetes, median (IQR)	13.0 (9.0–20.0)	12.0 (6.0–18.0)
HbA1c (mmol/mol)	72.2 (19.0)	78.4 (18.7)
Fasting glucose (mmol/L)	10.10 (3.76)	10.61 (3.02)
Insulin (U), median (IQR)	73.1 (41.3)	79.4 (70)
Insulin (U/kg), median (IQR)	0.72 (0.42)	0.75 (0.51)
Duration of insulin, median (IQR)	4.0 (2.0 to 6.2)	4 (2.5 to 8.0)
Other medications, n (%)		
Metformin	37 (82.2)	42 (93.3)
SU	10 (22.2)	16 (35.6)
GLP-1	14 (31.1)	3 (6.7)
Dipeptidyl peptidase IV inhibitors	5 (11.1)	7 (15.6)
SGLT-2 inhibitors	7 (15.6)	4 (8.9)
Thiazolidinediones	0 (0)	1 (2.2)
Oral antidiabetic medications, n	2.62 (0.94)	2.6 (0.83)
Blood pressure (mm Hg), mean (SD)		
Systolic	131.5 (16.1)	132.2 (17.6)
Diastolic	73.2 (9.1)	74.0 (12.7)
HDL cholesterol (mmol/L)	1.09 (0.30)	1.14 (0.35)
Triglycerides (mmol/L)	2.01 (2.14)	1.78 (1.41)
Hypertension, n (%)	32 (80.0)	35 (77.8)
CHD, n (%)	9 (22.5)	12 (26.7)
Smoking, n (%)	8 (17.8)	7 (15.6)
Statins, n (%)	40 (88.9)	41 (91.1)
Retinopathy, n (%)	19 (42.2)	16 (35.6)
Nephropathy, n (%)	6 (13.3)	12 (26.7)
Neuropathy, n (%)	10 (25.0)	17 (40.0)
Estimated glomerular filtration rate (mL/min/1.73 m^2^)	75.4 (17.1)	76.9 (21.4)
Quality of life (mm)	68.1 (19.9)	62.7 (19.4)

Data presented as mean (SD), n (%) or median (IQR) unless otherwise specified.

No significant difference between groups in any characteristics.

Nephropathy defined as having estimated glomerular filtration rate (eGFR) ≤60 mL/min/1.73m^2^.

CHD, Coronary Heart Disease; GLP-1, glucagon-like peptide-1 agonist; HbA1c, glycated hemoglobin; HDL, high-density lipoprotein; SGLT-2, sodium-glucose cotransporter-2 inhibitor; SU, sulfonylurea.

### Primary outcome

**Figure 1:**
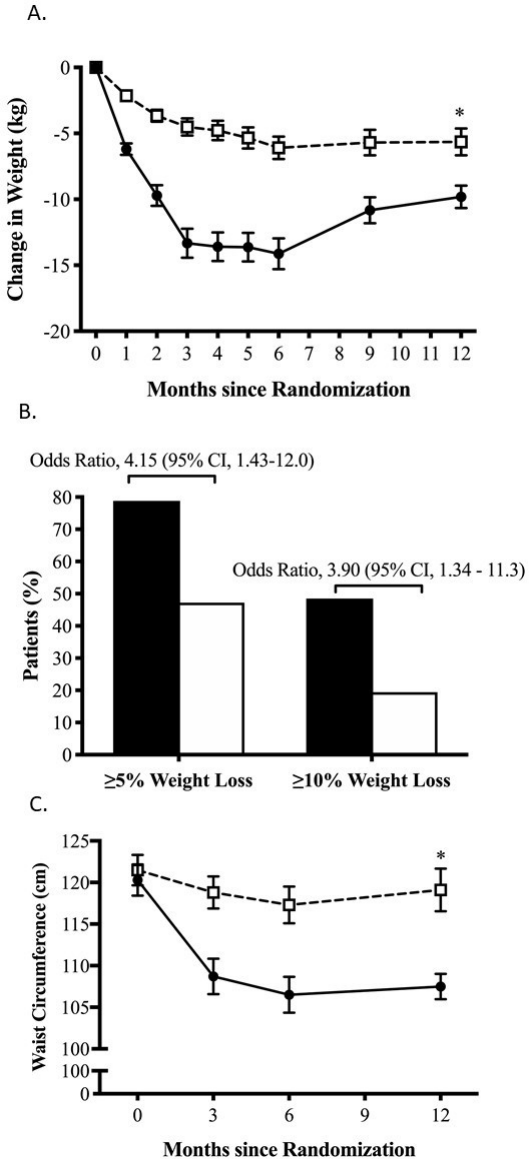
Body weight change comparing intervention and control over 12 months. (A) Line graph of mean body weight change from baseline with standard error of the mean (SEM). (B) The percentage of participants who achieved at least 5% or 10% weight loss from baseline at 12 months in the intervention and control groups. (C) Line graph of waist circumference change from baseline with SEM. P values, SEM and 95% CI calculated using adjusted mixed linear modeling. *P<0.05 between-group difference.

The mean reduction in body weight at 12 months was 9.8 kg (SD 4.9; 9.7% (SD 4.8) of initial weight) in the intervention group and 5.6 kg (SD 6.1; 5.8% (SD 6.5) of initial weight) (table 2) in the control group (adjusted difference −4.3 kg, 95% CI −6.3 to −2.3; p<0.0001; figure 1A). During the 12-week TDR LED phase, body weight in the intervention group reduced by 13.3 kg (SD 6.8) compared with 4.5 kg (SD 4.0) for the control group (p<0.0001), which continued to reduce during the food reintroduction phase to 14.1 kg (SD 6.9) vs 6.1 kg (SD 5.2), respectively. During subsequent 6 months, both groups regained weight (intervention 4.3 kg; control 0.5 kg; p<0.001). This did not change after sensitivity analysis (online supplementary table S1).

**Table 2:** Body weight, HbA1c and insulin usage outcomes

	Intervention	Control	Intervention effect	P value
			Estimate (95% CI)	
Weight (kg)				
Baseline	104.0±20.2	103.1±18.9		
Mean change at 12 months	−9.8±4.9	−5.6±6.1	−4.3 (−6.3 to −2.3)	<0.001
HbA1c (%)				
Baseline	8.75±1.74	9.32±1.71		
Mean change at 12 months	−0.43±1.01	−0.09±1.64	−0.56 (−1.17 to 0.05)	0.07
HbA1c (mmol/mol)				
Baseline	72.2±19.0	78.4±18.7		
Mean change at 12 months	−4.7±11.1	−1.0±17.9	−6.1 (−12.8 to 0.5)	0.07
Insulin dose (U)				
Baseline	73.1±41.3	79.4±61.0		
Mean change at 12 months	−47.3±36.4	−33.3±52.9	−18.6 (−29.2 to −7.9)	0.001
Insulin dose (U/kg)				
Baseline	0.72±0.42	0.75±0.51		
Mean change at 12 months	−0.45±0.36	−0.29±0.50	−0.16 (−0.26 to −0.06)	0.002
Stopping insulin (n/(%))				
Baseline	45 (100)	45 (100)	–	
Change at 12 months	13 (29)	3 (7)	–	0.001

Data presented as mean±SD.

Nephropathy defined as having estimated glomerular filtration rate (eGFR) ≤60 mL/min/1.73m^2^.

HbA1c, glycated hemoglobin.

At 12 months, weight loss of ≥5% of body weight occurred in 26 of 33 (79%) participants in the intervention group compared with 17 of 36 (47%) in the control group (OR 4.15, 95% CI 1.43 to 11.99). Weight loss ≥10% occurred in 16 of 33 (48%) in the intervention group compared with 7 of 26 (19%) in the control group (OR 3.90, 95% CI 1.34 to 11.38; figure 1B).

### Secondary outcomes

**Figure 2:**
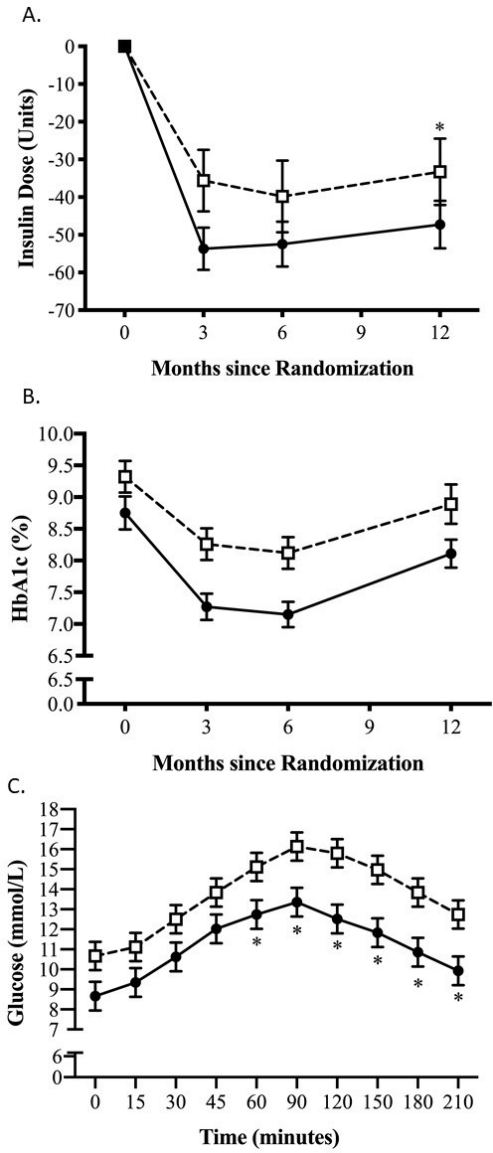
Insulin dose, glycated hemoglobin and postprandial plasma glucose change comparing treatment groups over 12 months. (A) Insulin dose reduction in units from baseline to 12 months in the intervention group and control group. (B) HbA1c change from baseline with standard error of the mean (SEM). (C) Postprandial plasma glucose concentrations at 12 months during the mixed meal tolerance test (MMTT). P values, SEM and 95%CI calculated using adjusted mixed linear modeling. *P<0.05 between-group difference.

At baseline, all participants were taking insulin for a median of 4.0 years (IQR 2, 7.25). At 12 months, 13 of 33 (39.4%) participants in the intervention group had discontinued insulin compared with 2 of 36 (5.6%) in the control group (p<0.001). At 12 months, there was a reduction in daily total insulin dose, reducing by 47.3 units (SD 36.4) (26.6 units/day (SD 27.2)) in the intervention group and 33.3 units (SD 52.9) (52.4 units/day (SD 41.6)) in the control group. The adjusted difference between the groups was −18.6 units (95% CI −29.2 to −7.9; p=0.001; figure 2A). At 12 months, participants in the intervention group were using significantly less sulfonylureas (SU) compared with the control (12.1% vs 33.3%, respectively, p*=*0.04, online supplementary table S2). Both metformin and gliptin usage was not significantly different between intervention and control groups at 12 months (metformin 78% vs 94.4%, p=0.054; gliptins 9.1% vs 19.4%, p=0.222) (table 2).

At 12 months, mean HbA1c fell by 0.43% (SD 1.01) (4.7 mmol/mol (SD 11.1)) in the intervention group and by 0.09% (SD 1.64) (1.0 mmol/mol (SD 17.9)) in the control group with an adjusted difference of −0.56% (95% CI −1.17 to 0.04; p=0.07) or −6.2 mmol/mol (95% CI −12.8 to 0.5; p=0.07; figure 2B). At 12 months, mean fasting plasma glucose was 8.7 mmol/L (SD 2.8) in the intervention and 10.7 mmol/L (SD 3.8) in the control group (−1.8 mmol/L, 95% CI −3.1 to −0.4; p=0.01; online supplementary figure S4A). The postprandial glucose response following the MMTT at 12 months was lower in the intervention 60–210 min following the test meal (figure 2C), with the glucose AUC_0–210_ also reflecting this with a significant adjusted difference of −2.12 mmol/L/min (95% CI −3.51 to −0.73; p=0.003; online supplementary figure S4B).

At 12 months, there was no difference in fasting and postprandial levels of C-peptide, or GLP-1 between the two groups (online supplementary table S4, figure S4C,D, respectively). At 6 months, the mean adjusted fasting C-peptide was lower in the intervention group than the control group (531.4 pmol/L vs 805.5 pmol/L, respectively), with a between-group difference of −274.1 pmol/L (95% CI −384.9 to −163.3; p=0.001).

The weight loss observed appeared to be mainly due to fat mass (FM) loss. FM loss was greater than lean mass in both groups (online supplementary figure S5A,B, respectively). FM loss in the intervention group was 7.0 kg (SD 4.3) compared with 4.3 kg (SD 4.4) in the control group (adjusted difference −2.4, 95% CI −4.1 to −0.7; p=0.006). Mean waist circumference reduced by 9.9 cm (SD 1.1) in the intervention group compared with 4.6 cm (SD 1.2) within the control group (adjusted difference −4.8, 95% CI −7.4 to −2.2; p<0.0001; figure 1C). At 12 months, reductions in anthropometric measurements from baseline including BMI and waist-to-hip ratio were greater with the TDR lifestyle intervention (online supplementary table S3).

At 12 months, CVD risk factors (low-density lipoprotein cholesterol, high-density lipoprotein cholesterol, triglycerides, systolic and diastolic blood pressure) did not differ between groups (online supplementary table S6). The between-group difference in CVD risk factors diminished over time (online supplementary table S6). The use of antihypertensive drugs and HMG-CoA reductase inhibitors was similar between the groups at baseline. Only two patients in the intervention group reduced antihypertensive drugs.

QoL improved by 11.1 points (SD 21.8) at 12 months in the intervention compared with 0.71 points (SD 19.4) in the control group (adjusted difference 8.6, 95% CI 2.0 to 15.2; p=0.01; online supplementary figure S6).

### Post hoc outcomes

Those who discontinued IT in the intervention group lost more weight than those who did not (12.2 kg (SD 4.1) vs 8.3 kg (SD 4.0); p=0.02). Furthermore, they had lower baseline HbA1c (7.9% (SD 0.8) vs 8.9% (SD 1.6)) (62.4 mmol/mol (SD 8.8) vs 74.4 mmol/mol (SD 17.7); p=0.03), lower baseline insulin dosage (43.7 units (SD 22.9) vs 93.4 units (SD 42.4); p<0.0001) and were mostly male (77% vs 30%; p=0.008, online supplementary table S5). There was a marked increase in 30 min postprandial increment in plasma C-peptide (30 min fasting) compared with baseline by 283.8 pmol/L (95% CI 137.2 to 430.4; p=0.001), despite no change in AUC_0–210_ C-peptide.

### Adverse events

Both groups reported easily managed mild or moderate adverse events (AEs) (online supplementary table S8). There was no significant difference in hypoglycemia between groups (adjusted incidence rate ratio 0.55, 95% CI 0.25 to 1.25; p=0.15), nor was there any difference observed during the TDR LED phase (online supplementary table S7). No hypoglycemia required assistance. The most frequent AEs in the intervention group were constipation (n=26), sensitivity to cold (n=23), flatulence (n=21), diarrhea (n=19) and dizziness (n=17).

Fourteen serious adverse events (SAE) occurred (nine control group and five intervention group). Only one SAE (an episode of postural hypotension) was thought to be intervention related. No deaths occurred during the study.

## Discussion

This is the first randomized clinical trial to demonstrate that a low-energy TDR-based lifestyle intervention safely induces weight loss, reduces insulin requirements, and improves QoL specifically in participants with long-standing type 2 diabetes and obesity receiving IT.

Traditionally, weight loss in those with type 2 diabetes has been considered to be challenging, with lower weight loss in patients on IT than those not on IT.^8^ In a review of weight loss interventions in non-IT participants with type 2 diabetes, 12-month pooled weight loss was 2.4–8.0 kg, while in the intensive lifestyle intervention arm of the Look AHEAD study, those on IT achieved a mean weight loss of 7.6%.^8^ Contrary to these findings, the weight loss achieved in the intervention group in our study (9.8 kg) was considerably greater and appeared to be mainly from FM.

In previous studies, patients with and without type 2 diabetes on a formula TDR LED program lost weight of 10.0–10.7 kg at 12 months, almost identical to our study, despite our participants being on IT.^10 25^ Furthermore, a larger proportion of participants in the intervention group achieved 5% and 10% weight loss with approximately a quadrupling of odds in achieving both these targets compared with the control group. Therefore, the TDR intervention provided an effective weight loss treatment in those with long-standing type 2 diabetes and obesity taking IT.

Postprandial glucose clearance after MMTT, particularly after 60 min, and fasting plasma glucose had greater improvement with the TDR intervention at 12 months, indicating enhanced insulin sensitivity, both peripherally and hepatically (figure 2C and online supplementary table S4). Unlike patients with shorter duration type 2 diabetes where weight loss is associated with decreases in fasting plasma insulin,^26^ we found no overall difference in fasting or postprandial plasma C-peptide or circulating GLP-1 at 12 months. In agreement with our findings, improvements in peripheral and hepatic insulin sensitivity have been observed previously following a VLED in those with type 2 diabetes who recently ceased IT.^12 27^

Although first phase insulin release was not formally measured, those who discontinued insulin had an early, 0–30 min (ΔC_30_), increase in postprandial C-peptide secretion, suggesting improved beta-cell function. This implies that the natural history of type 2 diabetes is more amenable to metabolic modification than expected, despite long-standing disease, and would benefit from formal measurement using gold standard, euglycemic hyperinsulinemic clamp.

There was a marked improvement in insulin usage and glycemic control by 12 months in the intervention group. Despite participants having type 2 diabetes for on average of 13 years, the average insulin usage in the intervention group fell to 27 units/day, with 39.4% of participants stopping insulin completely, compared with the control group who took 52 units/day, with only 5.6% stopping insulin. Reducing insulin dose and/or stopping it would be expected to reduce insulin-induced weight gain, hypoglycemia risk (online supplementary table S7) and the negative impact on QoL.^9^ Furthermore, there was a significant reduction in SU usage in the intervention group at 12 months, which was a result of medication reduction to prevent hypoglycemic episodes. It should be mentioned this may have impacted the weight loss achieved within the control group, due to the weight potentiating action of SU, however with there being no difference in hypoglycemic episodes (online supplementary table S7), the impact on body weight loss is likely to be minimal.

IT negatively affects QoL.^5 28^ Importantly, those in the intervention group reported a significantly improved QoL score at 12 months, while QoL was unchanged in the control group. The observed improvement was greater than reported following formula diets in non-IT type 2 diabetes (7.2 points) and bariatric surgery (9 points).^10 29^ The degree of weight loss, improved glycemic control and particularly reducing and stopping IT may have contributed to the improved QoL.^5^

Of clinical importance were those individuals who stopped IT. Minimizing IT, while improving glycemic control and body weight in type 2 diabetes, may reduce all-cause mortality,^30 31^ as there is a dose–response relationship between IT and all-cause mortality in type 2 diabetes.^32^ With concerns about the healthcare costs of insulin usage accompanying the expected increased type 2 diabetes prevalence,^33^ effective approaches, such as shown in our study, to stop IT are essential. Identifying those participants who might benefit most from such an intervention is of importance. From our study, those who stopped insulin were mainly men, had lower baseline insulin dosage and HbA1c and achieved a greater weight loss at 12 months.

### Strengths and limitations of this study

To our knowledge, this is the largest study to date in this complex patient population demonstrating that a low-energy TDR intervention can be used both effectively and safely. With support within the UK from NHS England for the use of LED in those recently diagnosed with type 2 diabetes, this study provides further evidence for promoting their use for type 2 diabetes, including those treated with insulin. The strengths of the study include the use of algorithms to reduce insulin dosage on starting the TDR intervention that effectively minimize both hyperglycemic and hypoglycemic episodes and should enable safe use of TDR into clinical practice. The participant population was typical of patients with long-standing type 2 diabetes, but the study benefited from having a more ethnically diverse group, a limitation found in previous studies using formula VLED and LED diets.^10^ The control group of this study was unique, in that the dietetic intervention was designed to achieve weight loss, which may have reduced the effects observed with the TDR intervention.

Our study has some limitations. Unlike recent studies of patients recently diagnosed with type 2 diabetes carried out in primary care, the interventions in our study were conducted in a secondary care outpatient setting allowing closer monitoring of the patients. Despite this, with the necessary support staff, a TDR intervention could be easily transferable to primary care in this population. Another important limitation was the reduced follow-up visits implemented after the initial 6 months, which reflected in changes in both HbA1c and body weight. After an initial HbA1c reduction at 6 months of the intervention of 1.39% (15.2 mmol/mol) compared with 0.72% (7.8 mmol/mol), in the control group, HbA1c levels were similar at 12 months (figure 2B). Despite no difference between groups, HbA1c was reduced in the intervention by clinically significant 0.43% (4.7 mmol/mol) compared with no reduction in the control group. Of note, the HbA1c reduction in the intervention group was accompanied by insulin reduction or cessation, which for this patient population has multiple benefits. Previous studies assessing the effect of VLED in patients with type 2 diabetes on IT have reported similar findings, with initial improvements in glycemic control followed by gradual deterioration,^13 14 27^ potentially representing the natural glycemic deterioration in those with long-standing type 2 diabetes. Other factors that could explain this deterioration are the weight regain, increase in waist circumference and FM between 6 and 12 months which may have had detrimental effects on insulin sensitivity and beta-cell function. The weight regain from 6 to 12 months may have diminished the earlier intervention effect. This weight regain may have been driven by multiple mechanisms, including a counter-regulatory effect on circulating gut hormone following weight loss affecting subjective hunger.^34^ With no change in either fasting or postprandial GLP-1 in either group, alterations of other gut hormones such as ghrelin or peptide YY may have contributed to weight regain. A comparable weight regain (4.4 kg) was, however, also seen following a similar reduction in patient contact in a recent primary care study employing an LED diet approach.^25^ This also highlights the importance of frequent contact to support weight loss maintenance following the initial TDR intervention and the need for additional strategies for weight maintenance, weight loss and glycemic control which could include GLP-1 receptor agonists, SGLT-2 inhibitors or continued/intermittent use of formula products.^10 35 36^

## Conclusions and implications to practice

This study confirms that a low-energy TDR intervention including behavior modification and physical activity can be used effectively to manage patients with type 2 diabetes and obesity receiving IT. At present there are very few effective treatments for those with long-standing type 2 diabetes apart from escalating pharmacotherapy or bariatric surgery. This study fills the current gap in knowledge not addressed by the Diabetes Remission Clinical Trial, which focused on those diagnosed with type 2 diabetes for less than 6 years who were not treated with insulin. Patients with long-standing type 2 diabetes and obesity on IT can, with sufficient weight loss achieved through a low-energy TDR intervention, reduce insulin burden and improve QoL. Maintenance strategies are required to ensure the preservation of the early beneficial effects of the TDR intervention.
