# Supplementary material for: Low-energy total diet replacement intervention in patients with type 2 diabetes mellitus and obesity treated with insulin: a randomized trial
Source: BMJ Open Diabetes Res Care. 2020 Jan 28;8(1):e001012. doi: 10.1136/bmjdrc-2019-001012 (PMC7039597; doi:10.1136/bmjdrc-2019-001012)
Supplement: Supplementary data [file bmjdrc-2019-001012supp001.pdf]

Supplementary Appendix

Table of Contents Page

Section	Page
Exclusion Criteria	2
Recruitment methods,	2
Informed consent	2-3
Withdrawal criteria	3
Telephone Consultations	3
Missing Data	3
Glucose Assay	3-4
Radioimmunoassay Details	4
Tables	5-11
Figures	12-17
References	17

Adrian Brown PhD, Anne Dornhorst DM, Barbara McGowan PhD, Omar Omar MSc, Anthony R Leeds MSc, Shahrad Taheri PhD, Gary Frost PhD

Exclusion Criteria

Exclusion criteria included: type 1 diabetes mellitus (based on clinical classification), insulin therapy for more than 10 years with a fasting circulating C-peptide of less than 600pmol, significant diabetes microvascular complications, a cardiovascular event within 6 months, left bundle branch block confirmed by electrocardiographic (ECG), estimated glomerular filtration rate (eGFR) of less than 30 mL/min/1.73m2, a condition precipitating fluid overload (e.g. New York Heart Association class III-IV congestive heart failure), mental incapacity, unwillingness and/or inability to understand and be able to complete the mental health questionnaires. Other exclusions included uncontrolled psychiatric disorder, uncontrolled depression, clinically diagnosed binge eating disorder, known or suspected substance use, concomitant medication use clinically deemed to affect metabolic rate and weight, participation in a weight management drug trial in the previous 3 months, pregnancy, lactating or planning pregnancy within study period, uncontrolled International Normalising Ratio (INR), uncontrolled epilepsy, lactose intolerance, severe musculoskeletal condition preventing walking, gout, active gallstones or known asymptomatic gallstones and clinically assessed hypoglycaemia unawareness. Glucagon-like peptide-1 (GLP-1) receptor agonists and sodium-dependent glucose co-transporters 2 (SGLT-2) inhibitors were ceased at the start of the study.

Recruitment methods

Database searches were completed using local systems and these lists were reviewed for appropriate subjects. Subjects were identified from local GP practices, community diabetes service and the Diabetes and Clinical Research Network (DRN and CRN) using the study inclusion criteria. Once an eligible subject was identified, an invitation pack was either sent by the general practice or given to potential subjects when they attended their clinic appointment. The invitation pack included an invitation letter, reply slip, self-addressed envelope and the invitation subject information sheet (PIS). Potential subjects who did not respond to the invitation letter were sent a letter or telephoned approximately 2 weeks after initial contact. The Diabetes Research Network (DRN) and Clinical Research Network (CRN) supported the recruitment process, as the study was on the National Institute for Health Research (NIHR) portfolio.

Potential subjects were identified from the Imperial College London Healthcare NHS Trust, Central London Community Healthcare NHS Trust, primary care within North West London, South London, the Hammersmith and Fulham Clinical Commissioning Group and the Clinical and Diabetes Research Network. Another additional site for recruitment and study visits was Guy's and St Thomas' NHS Foundation Trust.

It is planned for recruitment to happen over a 15-month period. With the patient's consent, their GP practice and diabetes specialists will be notified of their participation in the research.

Consent

Patients expressing an interest in the study will be given an appointment for a screening visit (Visit 1), a researcher will review the patient information sheet with the patient and informed consent will be taken. Signed consent will be obtained from patients. A signed copy of

informed consent form (ICF) will be given to the patient for their records, a copy will be sent to their GP and the final copy will be kept with the site file (SF).

Withdrawal Criteria

The patient has the right to withdraw their consent anytime without giving any reason. Their decision to withdraw from the trial will not affect their future legal and medical rights (16). The patient's reason for withdrawal will be recorded on the premature withdrawal form (PWF). This will be shown to patients before obtaining informed consent. Patients will be notified that if they choose to withdraw from the study, the investigative team will not proceed with further assessments and data collection, but their previously collected data will be used by the investigative team for research purposes. Patients will be given a withdrawal letter (appendix I) and a letter will also be sent to their general practitioner (GP) (appendix II). Also, the study will withdraw the patient if:

• The patient is unable to adhere to the study protocol and study requirements;
• There is a significant protocol deviation;
• The patient becomes pregnant during the study;
• Any new illness that affects their inclusion (exclusion criteria above);
• Continuing in this research is harmful to the patient health;
• The patient is lost to follow up;
• The trial cancels.

Telephone Consultations

Telephone consultations were scheduled during the week to allow for as much flexibility as possible to suit the needs of the subject. A member of the research team asked each subject to identify slots (days and times) that would be most suitable for them. Subjects had a clinical assessment, assessment of their adherence to the dietary changes, and queries to identify any issues with compliance or adverse events. Telephone consultations lasted up to 15 minutes and conducted every 2 weeks in between appointments a summary of the telephone consultation was made.

Missing Data

The impact of non-response and missing data at 12 months follow-up were examined in a sensitivity analyses. In order to avoid a loss in efficiency, missing values were imputed using multiple imputation by chained equations.[1] In this, 20 imputed datasets were created by replacing missing values with simulated values from a set of imputation models built from all potential prognostic and the outcome variable (weight loss). Last observation carried forward and analysis of the completers subset only was also performed as part of the sensitivity analyses

Glucose Assay

Glucose was determined after enzymatic oxidation in the presence of glucose oxidase. The hydrogen peroxide formed reacts, under catalysis of peroxidase with phenol and 4-aminophenazone to form a red-violet quinoneimine dye (Raba, 1995).

Glucose analysis was performed in house using Randox Glucose Assay Kit. Reagent (1000µl) was added to an Eppendorf and 10µl of sample was added and vortexed. The mixture was

incubated for 10 minutes at 37°C. Next 200µl was pipetted into a 96 microwell plate and a spectrometry was used to assess the glucose concentrations of the sample. The machine was set up at 37°C with a wavelength of 540nm. Glucose analysis was carried out in separate assays according to subject with mean percentage coefficient of variance of 2.7%.

Radioimmunoassay

Radioimmunoassays were performed in the Department of Medicine at Imperial College London. The principle of RIA is based upon the competitive binding between radiolabeled antigen and unlabeled antigen for specific high-affinity antibody sites. A fixed concentration of radiolabeled tracer antigen is incubated with a constant dilution of antiserum. This limits the number of antigen binding sites on the antibody. When unlabeled antigen is added, there is a competition between the labelled tracer for the binding sites on the antibody. With increasing concentrations of unlabeled antigen, the amount of radiolabeled antigen bound to the antibody site decreases.

After centrifugation, the bound (pellet) and unbound (supernatant) are separated and the amount of radioactivity is counted on a gamma counter.

As standard curve is set up with increasing concentrations of standard unlabeled antigen which allows for the quantification of the concentration of antigen in the unknown sample by interpolation. Insulin analysis was carried out in three parts with percentage coefficient of variance (% CV) of 4.7%, 2.6%, 8.8%

The insulin RIA was carried out using a Millipore Human Insulin Specific RIA Kit (Millipore Corporation, Billerica, USA) according to the manufacturer's protocol.

The assay was incubated for 20-24 hours at room temperature before separation of the free from bound antibody labelled by secondary precipitating reagent. Tubes were drained and the radioactivity of the pellets was counted using a gamma scintillation counter (LB2111 MultiCrystal Gamma Counter, Berthold Technologies, Bad Wildbad, Germany) for 60 seconds.

Supplementary Tables

Table S1: Sensitivity analysis of primary outcome

	LOCF		Multiple imputation		Completers only	
	Intervention (n=45)	Control (n=45)	Intervention (n=45)	Control (n=45)	Interventon (n=33)	Control (n=36)
Unadjusted weight change from baseline	-9.4 (6.9)	-4.7 (5.9)	-8.8 (6.2)	-4.8 (6.5)	-9.8 (4.9)	-5.6 (6.1)
Difference between groups	-4.2 (-6.3 to -2.0)		-3.2 (-5.6 to -0.8)		-4.3 (-6.5 to -2.1)	
P-value	<0.001		0.009		<0.001	

Data represents mean plus standard deviations *P* values calculated using adjusted mixed linear modelling. LOCF, last observation carried forward

Brown A, *et al. BMJ Open Diab Res Care* 2020; 8:e001012. doi: 10.1136/bmjdrc-2019-001012

Table S2: Medication usage at baseline and 12 months.

Medication	Baseline			12 months		
	Intervention (n=45)	Control (n=45)	P-value	Intervention (n=33)	Control (n=36)	P-value
**Metformin, n (%)**	37 (82.2)	42 (93.3)	0.108	26 (78.8)	34 (94.4)	0.054
**Sulfonylureas, n (%)**	10 (22.2)	16 (35.6)	0.163	4 (12.1)	12 (33.3)	0.037
**GLP-1, n (%)**	14 (31.1)	3 (6.7)	0.003	-	-	
**Gliptins, n (%)**	5 (11.1)	7 (15.6)	0.535	3 (9.1)	7 (19.4)	0.222
**SGLT-2, n (%)**	7 (15.6)	4 (8.9)	0.334	-	-	
**Glitazones, n (%)**	0 (0)	1 (2.2)	0.315	0 (0)	0 (0)	-

Abbreviation: GLP-1, glucagon like peptide-1; SGLT-2, sodium glucose co-transporter-2 inhibitor; %, percentage, n, number

Table S3: Time course of anthropometric data at baseline, 3, 6 and 12 months comparing intervention in comparison with control in subjects who completed 12 months

	Baseline	3 months	6 months	12 months
**Weight (kg)**				
**Intervention**	104.0±20.2	89.1±17.2	88.3±17.5	89.3±12.9
**Control**	103.1±18.9	100.1±20.2	98.4±21.6	99.4±22.8
**BMI (kg/m^2)**				
**Intervention**	36.6±5.1	31.7±4.8	31.4±4.7	32.0±3.8
**Control**	36.8±5.3	35.6±5.7	35.2±6.3	35.5±6.8
**Fat Mass (kg)**				
**Intervention**	41.2±16.0	31.6±14.8	30.0±15.5	30.9±8.4
**Control**	42.1±11.2	38.7±12.0	38.1±13.3	39.2±13.0
**Lean Mass (kg)**				
**Intervention**	61.4±12.4	57.4±10.3	58.2±10.5	58.6±11.0
**Control**	60.6±11.6	59.8±12.1	58.7±12.2	58.8±12.6
**Waist Circumference (cm)**				
**Intervention**	120.3±12.7	108.7±13.1	106.5±12.7	107.5±8.8
**Control**	121.5±12.4	118.8±12.0	117.3±13.4	119.1±15.2
**Hip Circumference (cm)**				
**Intervention**	120.7±12.1	111.3±10.9	109.4±10.1	109.7±8.6
**Control**	122.0±13.2	119.7±13.5	117.9±14.4	119.5±15.7
**WHR**				
**Intervention**	1.00±0.06	0.98±0.06	0.97±0.06	0.98±0.05
**Control**	1.00±0.06	1.00±0.06	0.99±0.07	1.00±0.07

Abbreviations: kg, kilogram; kg/m^2, kilogram per meter squared; cm, centimeter; BMI, Body Mass Index; WHR, waist-to-hip ratio

Table S4: Fasting and Post Prandial Glycemic control usage comparing intervention with control.

Brown A, *et al. BMJ Open Diab Res Care* 2020; 8:e001012. doi: 10.1136/bmjdrc-2019-001012

	**Intervention**		**Intervention effect**	
	Intervention	Control	Estimate (95% CI)	*P* value
**Fasting Glucose (mmol/mol)**				
Baseline	10.10 (3.76)	10.61 (3.02)		
Mean Change at 12 months	-1.21 (2.41)	0.31 (4.46)	-1.75 (-3.12 to -0.38)	0.01
**Fasting C-peptide (pmol)**				
Baseline	658.3 (407.6)	639.9 (344.1)		
Mean Change at 12 months	-60.4 (283.9)	-11.6 (304.8)	-62.0 (-177.0 to 53.0)	0.29
**Fasting GLP-1 (pmol)**				
Baseline	79.7 (43.5)	74.2 (23.6)		
Mean Change at 12 months	-7.1 (33.2)	-3.1 (22.1)	-1.2 (-12.0 to 9.7)	0.83
**Glucose AUC (mmol/L/min)**				
Baseline	13.52 (4.22)	13.99 (2.93)		
Mean Change at 12 months	-1.52 (2.87)	0.41 (4.24)	-2.12 (-3.51 to -0.73)	0.003
**C-peptide AUC (pmol/min)**				
Baseline	1104 (659)	1009 (497)		
Mean Change at 12 months	-52 (393)	25 (338)	-64 (-205 to 78)	0.38
**GLP-1 AUC (pmol/min)**				
Baseline	86.6 (32.5)	87.4 (24.6)		
Mean Change at 12 months	8.7 (23.9)	0.14 (26.1)	8.4 (-3.3 to 20.1)	0.16

P values and 95% CI calculated using adjusted mixed linear modelling. Abbreviations: CI, confidence intervals; mmol/mol, millomoles per mole; pmol, picomoles; %, percentage; mmol/L/min, millomoles per litre per minute; pmol/min, picomoles per minute; GLP-1, glucagon-like peptide-1; AUC, area under the curve

Brown A, *et al. BMJ Open Diab Res Care* 2020; 8:e001012. doi: 10.1136/bmjdrc-2019-001012

Table S5: Characteristics of those who stopped insulin at 12 months vs. those who did not stop (intervention group only)

Characteristics	Stopped Insulin (n=13)	Did not stop Insulin (n=20)	***P***-value
Age at randomization, yrs, median (IQR)	63.1 (54.1, 65.7)	59.3 (49.8, 62.9)	0.269
Sex, n (%)			0.008
Male	10 (76.9)	6 (30.0)	
Female	3 (23.1)	14 (70.0)	
Duration of Diabetes, yrs, median (IQR)	13.0 (9.0, 20.0)	15.0 (10.0, 19.5)	0.75
HbA1c (%), mean (SD)	7.9 (0.8)	8.9 (1.6)	0.03
HbA1c (mmol/mol), mean (SD)	62.4 (8.8)	74.4 (17.7)	0.03
Insulin (U), mean (SD)	43.7 (22.9)	93.4 (42.4)	<0.001
Insulin (U/kg), mean (SD)	0.43 (0.22)	0.97 (0.42)	<0.001
Number of oral antidiabetic medications, mean (SD)	2.6 (1.0)	2.5 (1.1)	0.65
Weight Loss at 12 months kg, (SD)	12.2 (4.1)	8.3 (4.8)	0.02
Weight regain 6 to 12 months (kg), mean (SD)	4.0 (4.7)	2.7 (2.7)	0.32
Fasted glucose, mmol/L mean, (SD)	8.9 (2.4)	10.9 (4.0)	0.11

Abbreviations: IQR, interquartile range; %, percentage; n, number; mmol/mol millimoles per mole; yrs, years; U, units; U/kg, units per kilogram; mmol/L – millimoles per liter; kg, kilogram; HbA1c, glycated hemoglobin; SD, standard deviation

Brown A, *et al. BMJ Open Diab Res Care* 2020; 8:e001012. doi: 10.1136/bmjdrc-2019-001012

Table S6: Surrogate markers of cardiovascular disease comparing intervention with control

	**Intervention**		**Intervention effect**	
	Intervention	Control	Estimate (95% CI)	*P* value
**Systolic BP (mmHg)**				
**Baseline**	131.5±16.1	132.2±17.6		
**Mean Change at 12 months**	-7.7±15.0	-7.7±13.3	0.8 (-4.6 to 6.3)	0.76
**Diastolic BP (mmHg)**				
**Baseline**	73.2±9.1	74.0±12.7		
**Mean Change at 12 months**	-5.8±9.5	-3.5±9.0	-0.62 (-4.5 to 3.3)	0.76
**LDL-Cholesterol (mmol/L)**				
**Baseline**	2.19±0.92	2.20±0.96		
**Mean Change at 12 months**	0.13±0.68	-0.05±0.70	0.20 (-0.08 to 0.47)	0.16
**HDL-Cholesterol (mmol/L)**				
**Baseline**	1.09±0.30	1.14±0.35		
**Mean Change at 12 months**	0.14±0.23	0.07±0.22	0.05 (-0.03 to 0.13)	0.21
**Triglycerides (mmol/L)**				
**Baseline**	2.01±2.14	1.78±1.41		
**Mean Change at 12 months**	-0.57±1.99	0.12±0.80	-0.36 (-0.83 to 0.11)	0.14
**eGFR (ml/min per 1.73m2)**				
**Baseline**	75.4±17.1	76.9±21.4		
**Mean Change at 12 months**	2.4±9.6	-2.0±15.1	4.2 (-0.2 to 8.5)	0.06
**ALT (IU/L)**				
**Baseline**	29.5±13.5	28.7±17.3		
**Mean Change at 12 months**	-7.7±9.7	-5.1±10.6	-2.4 (-6.4 to 1.5)	0.23
**Alk Phos (IU/L)**				
**Baseline**	76.5±21.3	84.5±25.9		
**Mean Change at 12 months**	-2.0±9.3	-2.1±15.7	-2.1 (-8.1 to 3.9)	0.49
**Bilirubin (µmol/L)**				
**Baseline**	9.47±5.11	9.04±4.91		
**Mean Change at 12 months**	1.32±4.34	-0.17±3.44	1.44 (-0.36 to 3.25)	0.12
**hsCRP (mg/L)**				
**Baseline**	5.77±7.65	6.22±6.01		
**Mean Change at 12 months**	-2.72±8.19	-2.23±3.33	-0.23 (-2.24 to 1.80)	0.83

Data represents mean plus standard deviations p values calculated using adjusted mixed linear modelling. Abbreviations: LDL, low density lipoprotein; HDL; high density lipoprotein; eGFR, estimated Glomerular Filtration Rate; ALT, Alanine transaminase; ALP, Alkaline Phosphatase (ALP), hsCRP, High-sensitive C-reactive protein; mmHg, millimeter of mercury; mmol/L – millimoles per liter; IU/L, international units per liter; µmol/L, micromole per liter; mg/L, milligram per liter; CI, confidence intervals

Table S7: Self-reported frequency of hypoglycemic episodes between subject in the intervention and control.

**Hypoglycemic Episodes**	**Intervention (n, %)**	**Control (n, %)**
**0**	11 (24.4)	11 (24.4)
**1-2**	12 (26.7)	11 (24.4)
**3-4**	3 (6.7)	5 (11.1)
**5-6**	6 (13.3)	2 (4.4)
**7-8**	6 (13.3)	3 (6.7)
**9-10**	3 (6.7)	2 (4.4)
**10+**	7 (15.6)	14 (31)

Abbreviations: Data are presented as reported cases no. (%), Abbreviations: n, number; %, percentage

Table S8: Self-reported adverse events among the subject in the intervention and control at 24 weeks.

Side Effect	Frequency of side effect in intervention (n, %)	Frequency of side effect in control (n, %)
**Constipation**	26 (57.8)	13 (28.9)
**Sensitivity to cold**	23 (51.1)	1 (2.2)
**Flatulence**	21 (46.7)	2 (4.4)
**Diarrhoea**	19 (42.2)	7 (15.6)
**Dizziness**	17 (37.8)	7 (15.6)
**Bad breath**	16 (35.6)	0 (0)
**Fatigue**	14 (31.1)	16 (35.6)
**Dry Skin**	14 (31.1)	0 (0)
**Mood changes**	13 (28.9)	14 (31.1)
**Sleeplessness**	11 (24.4)	10 (22.2)
**Headaches**	11 (24.4)	6 (13.3)
**Toothache**	9 (20.0)	3 (6.7)
**Skin Irritation**	8 (17.8)	4 (8.9)
**Joint Pain**	8 (17.8)	11 (24.4)
**Nausea**	8 (17.8)	6 (13.3)
**Depressive tendencies**	7 (15.6)	11 (24.4)
**Anxiety**	7 (15.6)	13 (28.9)
**Back Pain**	7 (15.6)	7 (15.6)
**Hair loss**	7 (15.6)	1 (2.2)
**Abdominal Pain**	6 (13.3)	5 (11.1)
**Perianal Itching**	5 (11.1)	0 (0)
**Cramps**	4 (8.9)	2 (4.4)
**Allergic Rash**	4 (8.9)	1 (2.2)
**Sciatic Pain**	4 (8.9)	5 (11.1)
**Influenza**	4 (8.9)	3 (6.7)
**Heart Burn**	3 (6.7)	2 (4.4)
**Eczema**	3 (6.7)	1 (2.2)
**Redness**	3 (6.7)	4 (8.9)
**Swollen Joints**	2 (4.4)	5 (11.1)
**Hives**	2 (4.4)	0 (0)
**Epigastric pain**	1 (2.2)	5 (11.1)
**Vomiting**	1 (2.2)	3 (6.7)
**Taste alterations**	1 (2.2)	0 (0)
**Bilary Pain**	0 (0)	0 (0)

Abbreviations: Data are presented as reported cases no. (%); Abbreviations: n, number; %, percentage

Supplementary Figures

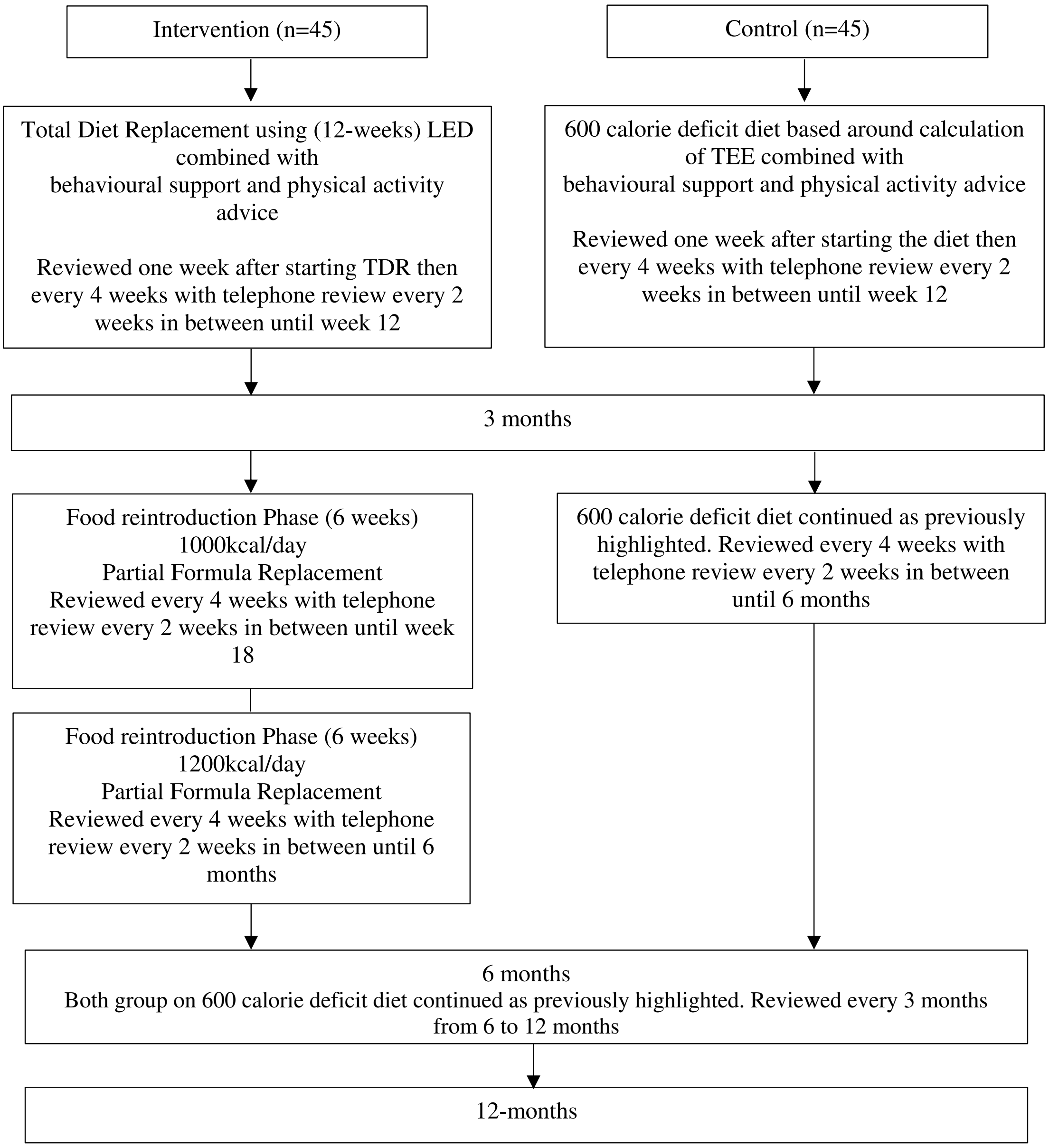

Figure S1: Intervention and Control

Abbreviations: LED Low Energy Diet; TDR, Total Diet Replacement; kcal/day, kilocalories per day; n, number

Brown A, *et al. BMJ Open Diab Res Care* 2020; 8:e001012. doi: 10.1136/bmjdrc-2019-001012

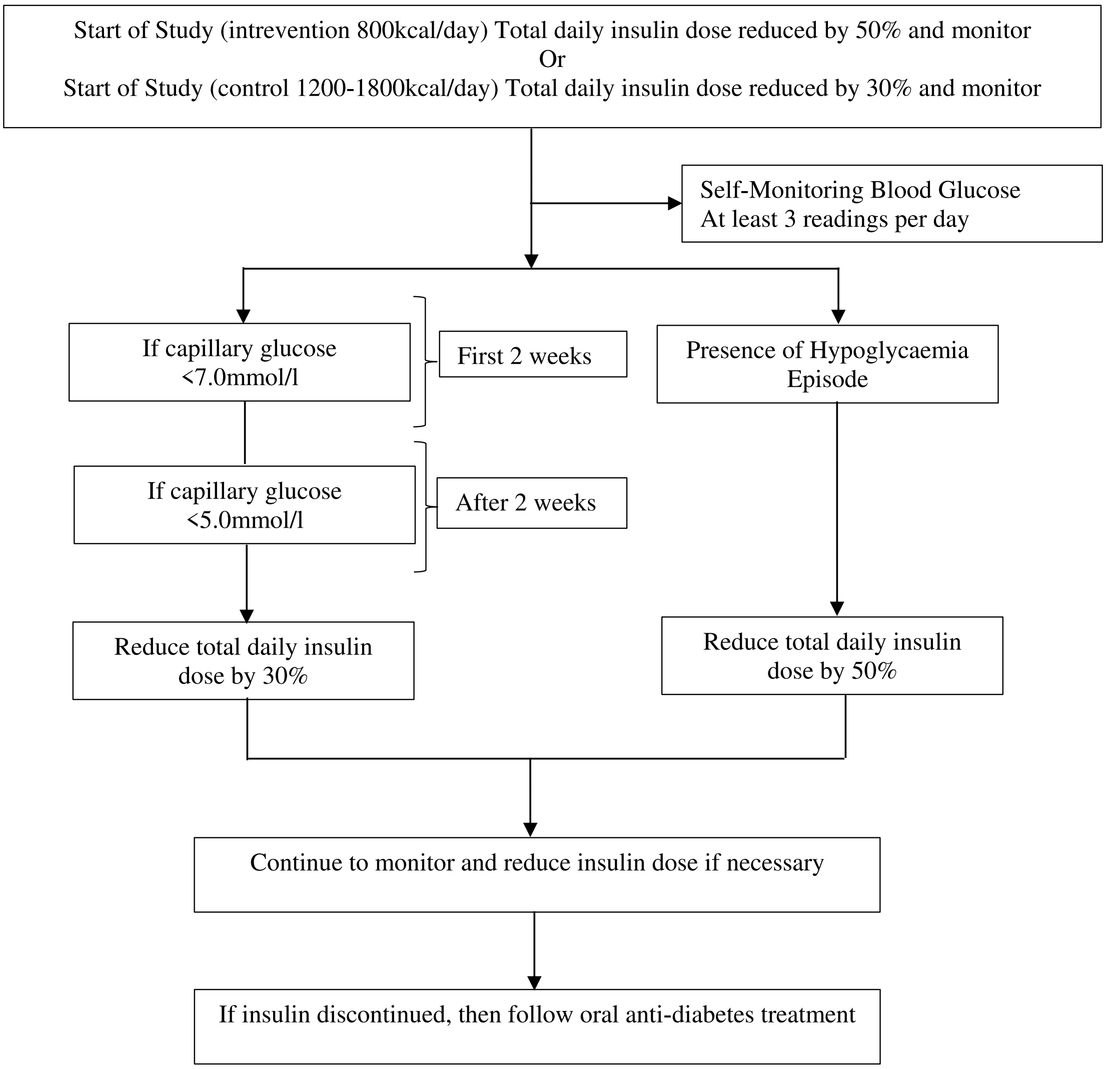

Figure S2: Insulin Titration algorithm for intervention and control

NOTE: If fasting capillary glucose rises to ≥ 12 mmol/l on 3 consecutive days, then insulin was up-titrated as clinically indicated. Abbreviations: kcal/day, kilocalories per day; %, percentage; mmol/L – millimoles per liter

Brown A, *et al. BMJ Open Diab Res Care* 2020; 8:e001012. doi: 10.1136/bmjdrc-2019-001012

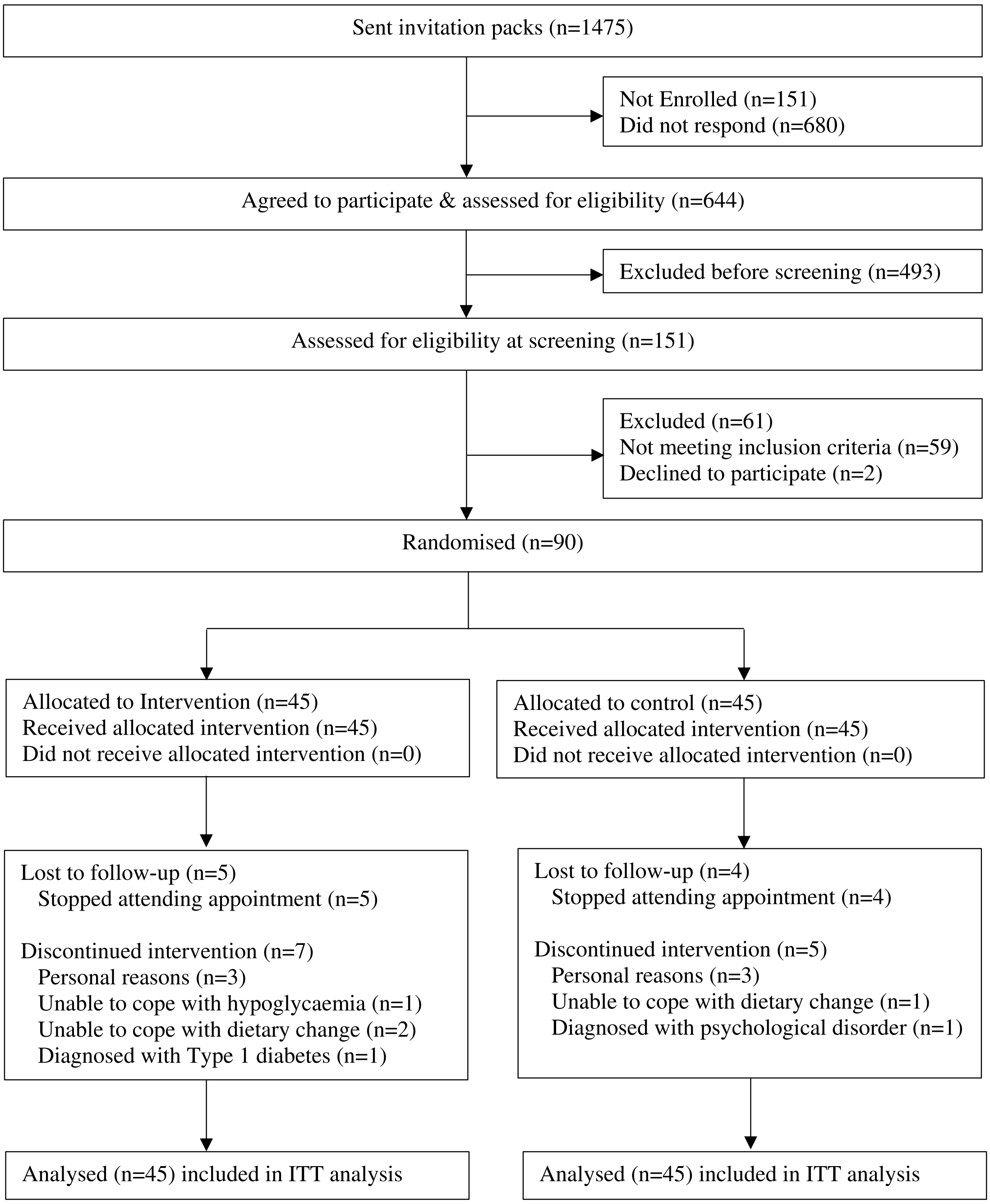

Figure S3: CONSORT Flow

Abbreviations: ITT = intention to treat, n, number

Brown A, *et al. BMJ Open Diab Res Care* 2020; 8:e001012. doi: 10.1136/bmjdrc-2019-001012

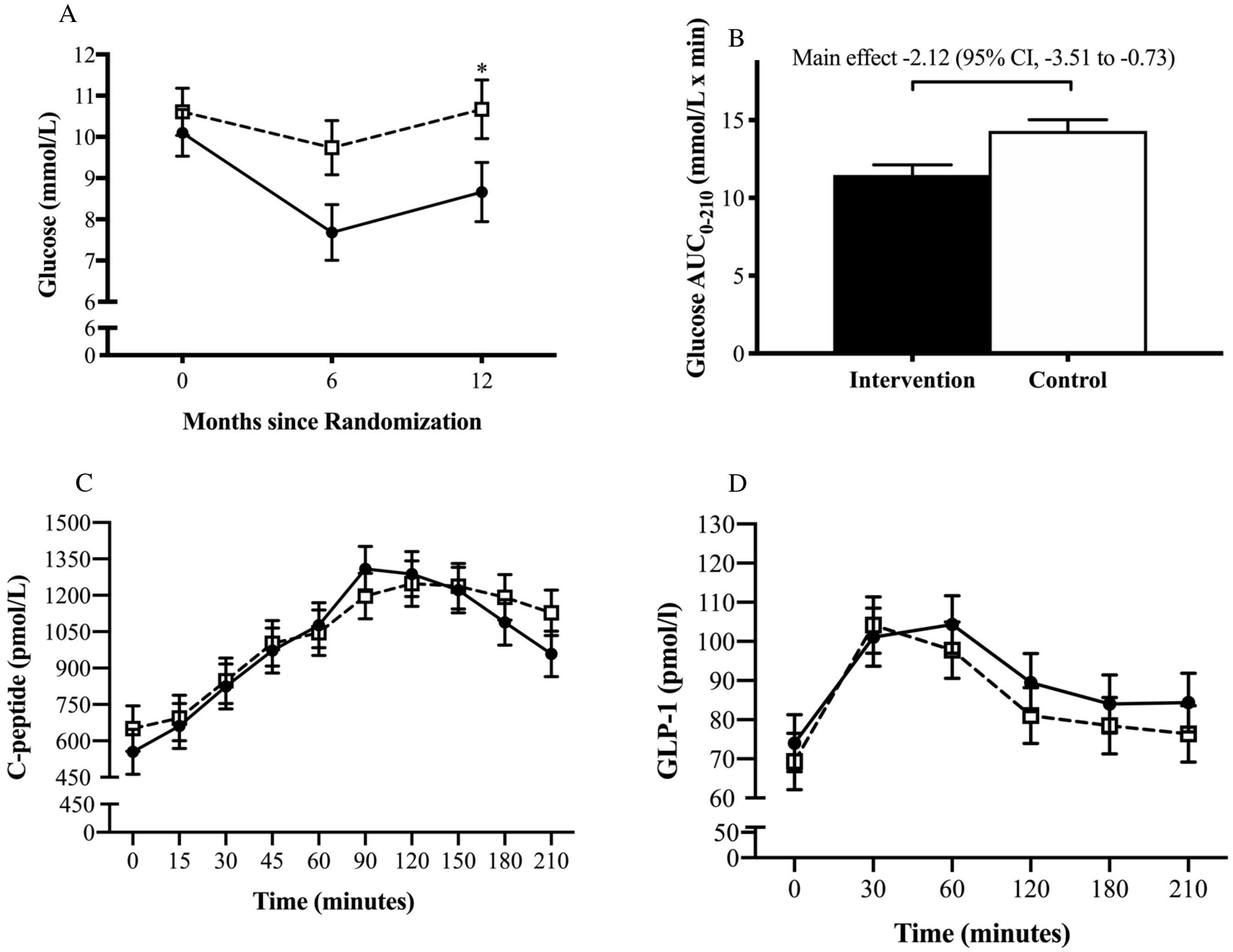

Figure S4: Fasting Plasma Glucose & Plasma AUC_{0-210} glucose concentrations comparing intervention and control at 12 months.

A shows line graph represents mean fasting glucose with standard error bars

B shows mean plasma AUC $_{0-210}$ glucose with standard errors.

C shows postprandial C-peptide at 12 months concentrations at 12 months during the MMTT.

D shows postprandial GLP-1 at 12 months concentrations at 12 months during the MMTT.

P values, SEM and 95% CI calculated using adjusted mixed linear modelling *p<0.05

Black circles = intervention; white square = control; Figure B, black bar = intervention, white bar = control

Abbreviations AUC, area under the curve; CI, confidence intervals; mmol/L, millimoles per liter; mmol/L x min, millimoles per liter per minute; pmol/L, picomole per liter

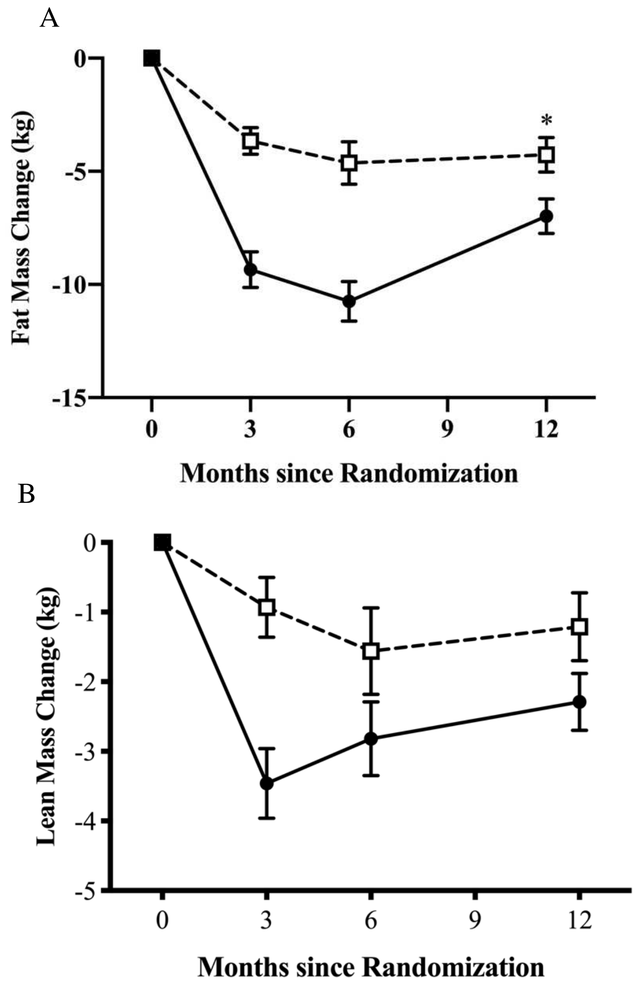

Figure S5: Lean Mass change and Fat Mass Change comparing intrevention and control over 12 months

A shows fat mass change from baseline to 12 months with standard errors.
B shows lean mass change from baseline to 12 months with standard errors.
P values, SEM and 95% CI calculated using adjusted mixed linear modelling *p<0.05 between group difference.
Black circles = intervention; white square = control;
Abbreviations: kg, kilogram; CI, confidence intervals

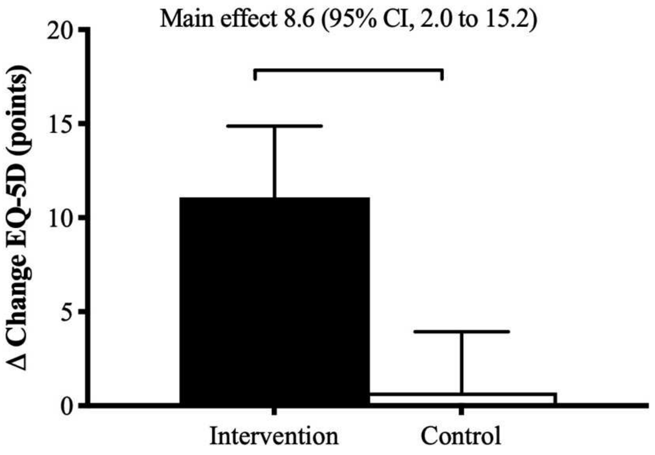

Figure S6: Quality of life change comparing intervention and control over 12 months
P values, SEM and 95% CI calculated using adjusted mixed linear modelling.
Black bar = intervention; white bar = control
Abbreviations: EQ-5D EuroQoL 5 dimensions; CI, confidence intervals; **Δ**, delta; points.

References

1. Royston P. Multiple imputation of missing values: update of ice The Stata Journal 2005;5:527-36.
